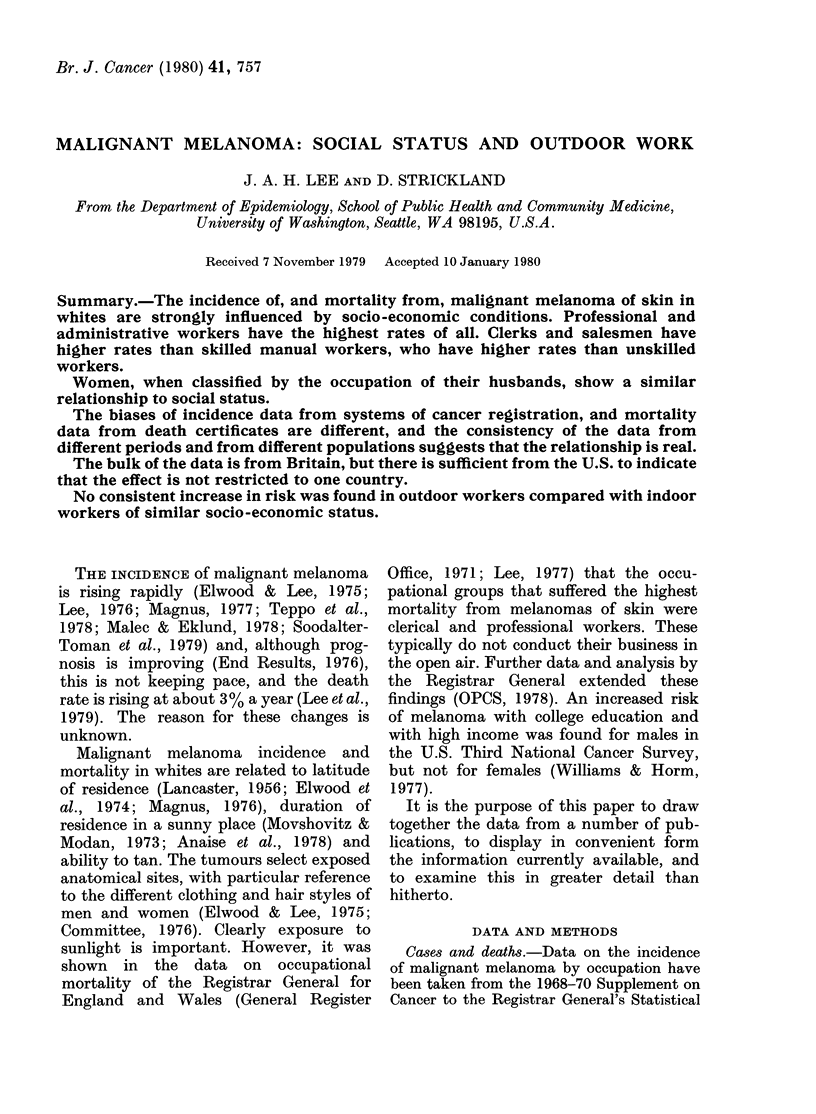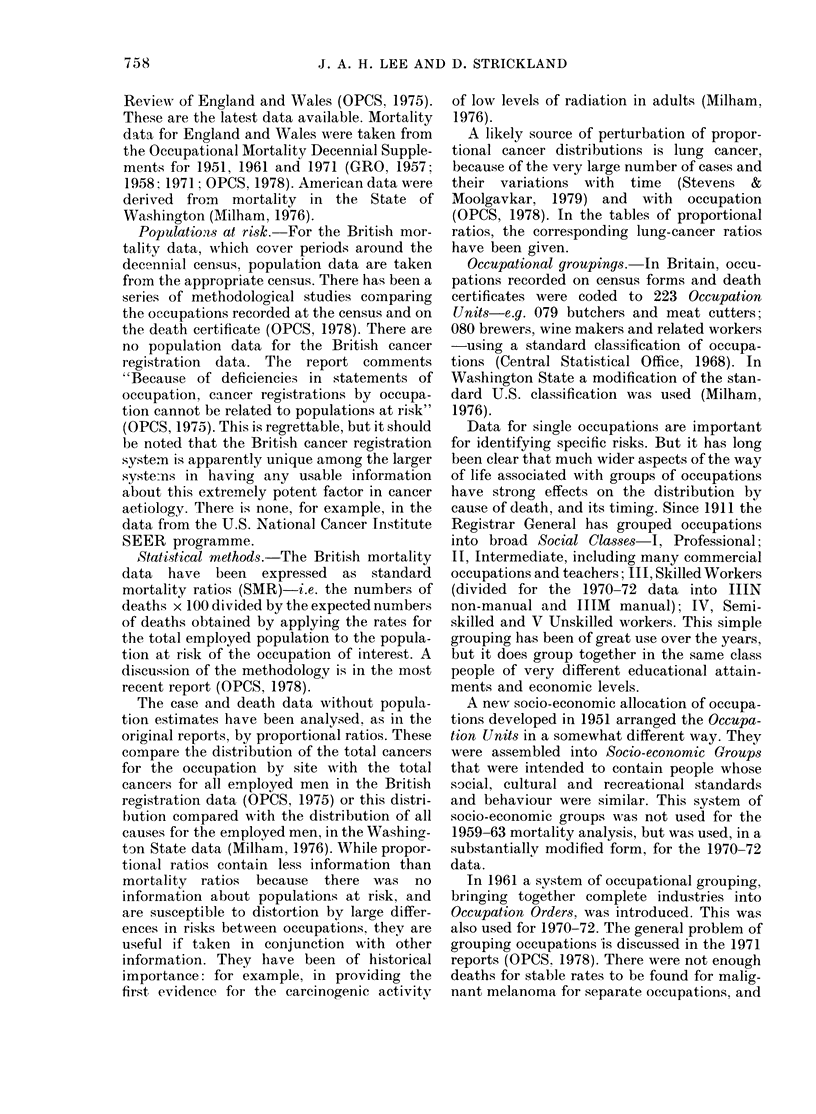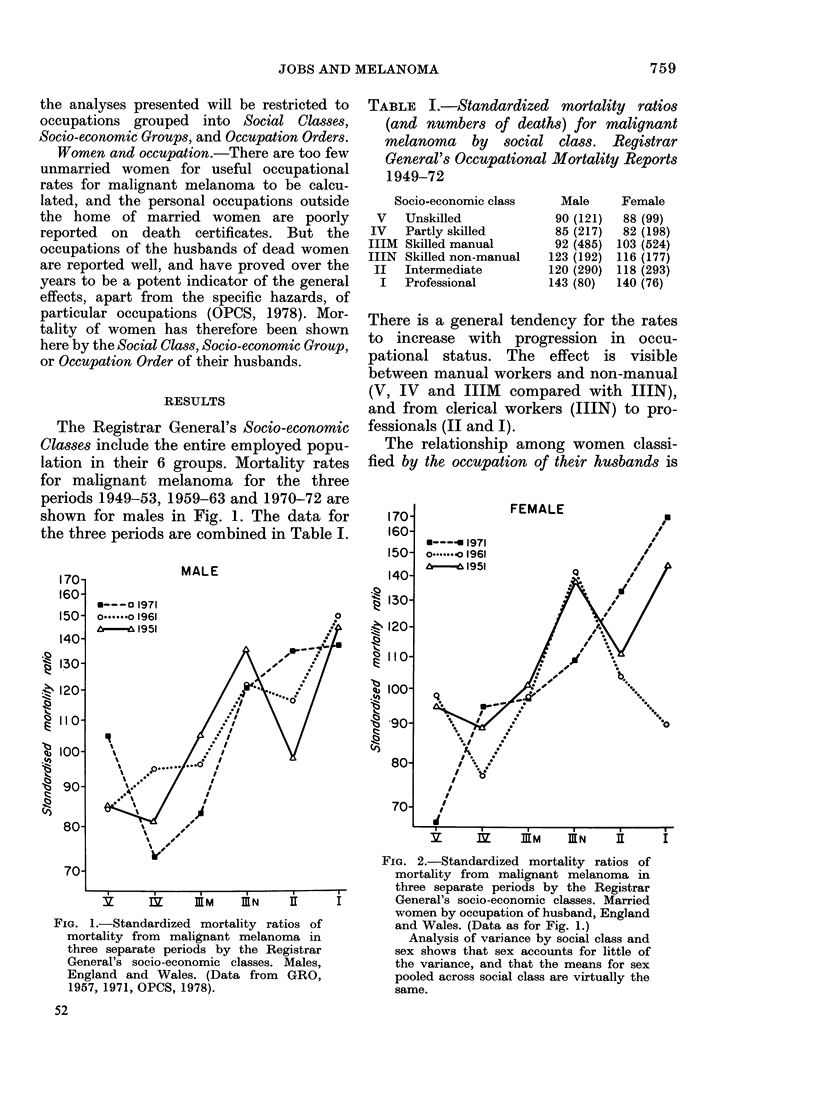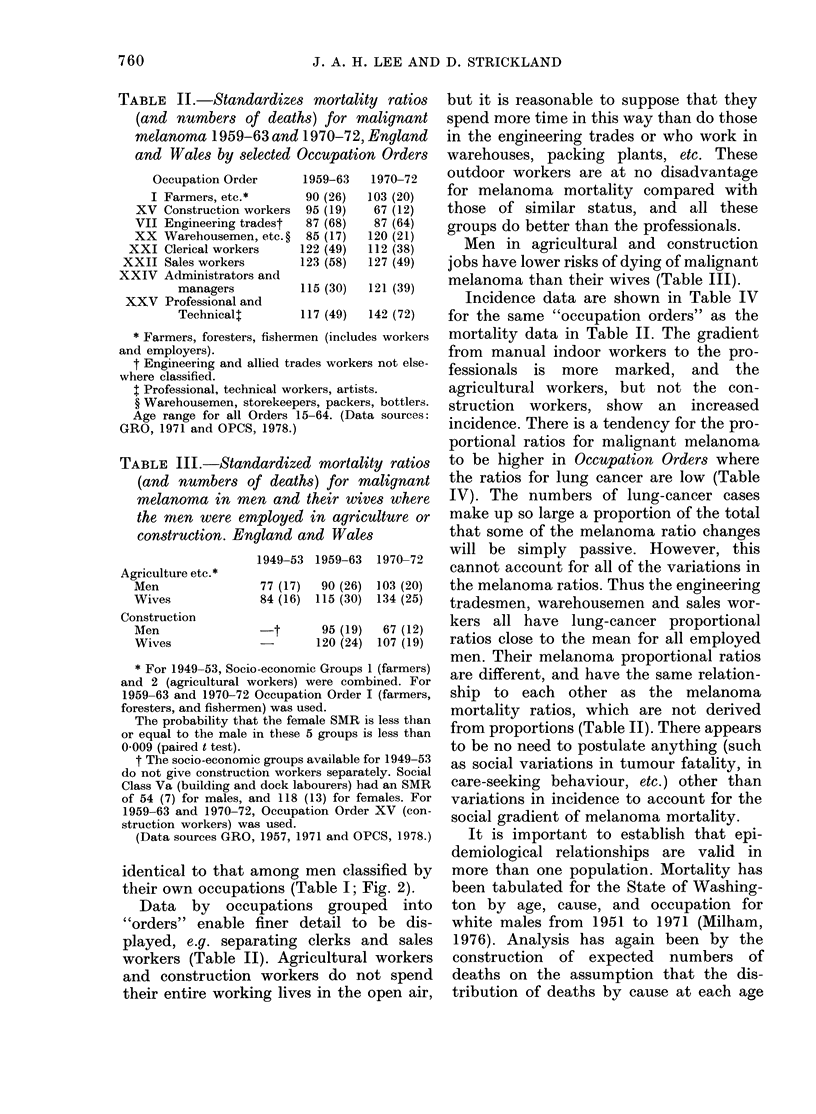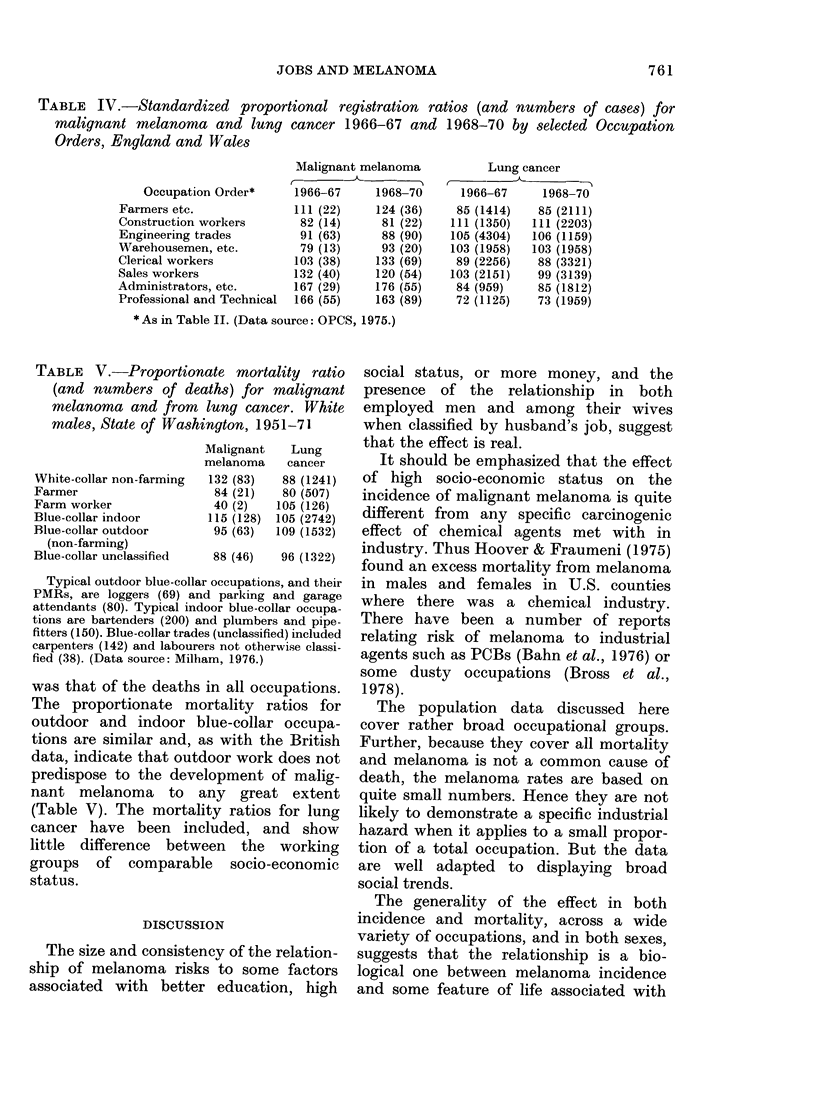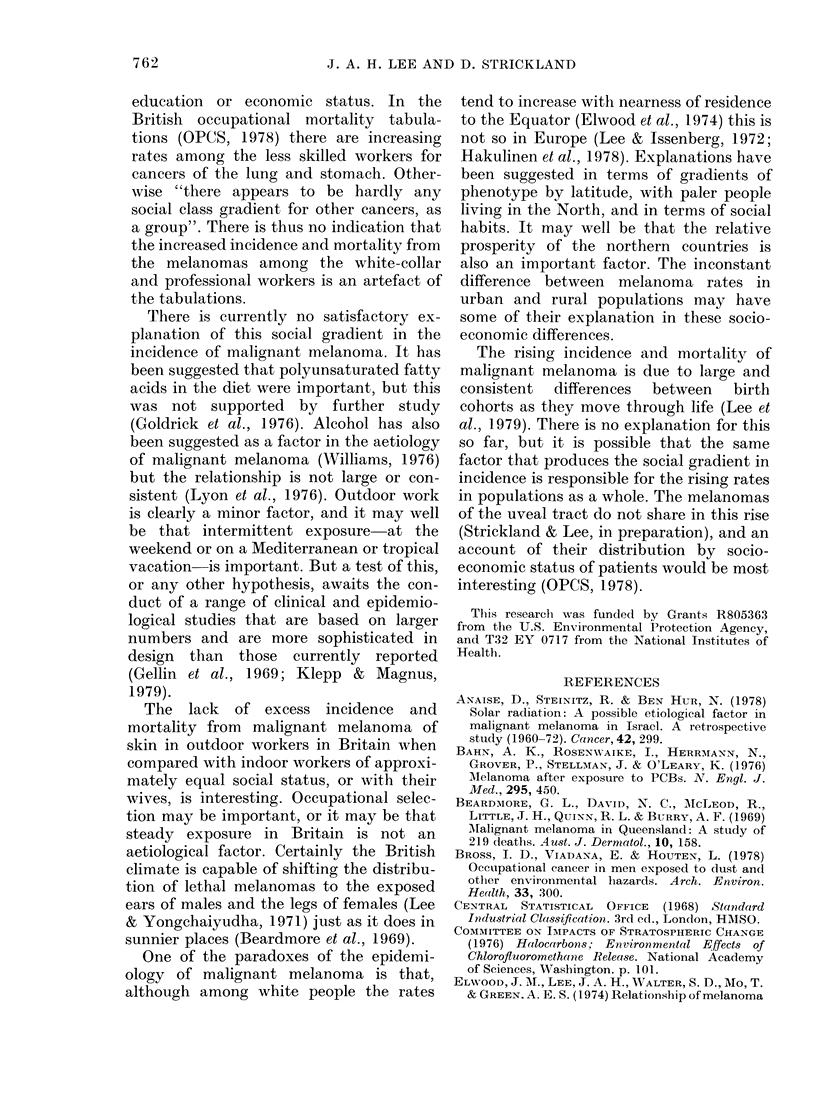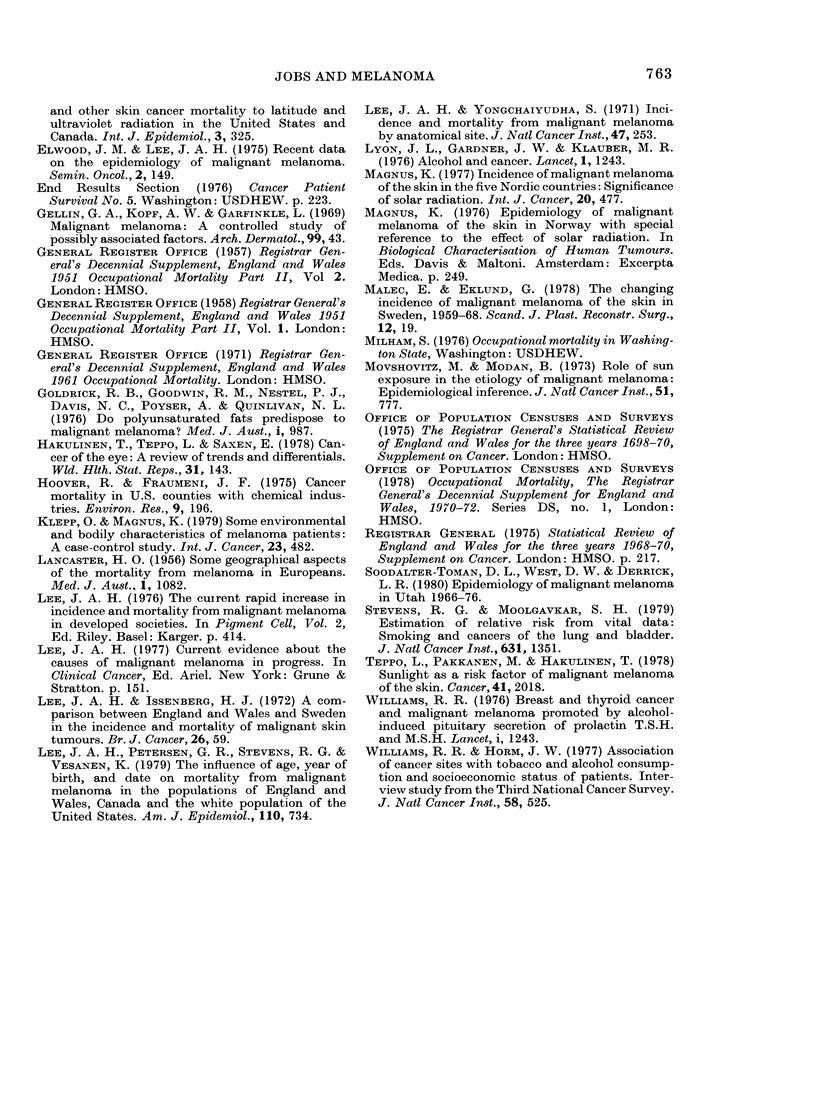# Malignant melanoma: social status and outdoor work.

**DOI:** 10.1038/bjc.1980.138

**Published:** 1980-05

**Authors:** J. A. Lee, D. Strickland

## Abstract

The incidence of, and mortality from, malignant melanoma of skin in whites are strongly influenced by socio-economic conditions. Professional and administrative workers have the highest rates of all. Clerks and salesmen have higher rates than skilled manual workers, who have higher rates than unskilled workers. Women, when classified by the occupation of their husbands, show a similar relationship to social status. The biases of incidence data from systems of cancer registration, and mortality data from death certificates are different, and the consistency of the data from different periods and from different populations suggests that the relationship is real. The bulk of the data is from Britain, but there is sufficient from the U.S. to indicate that the effect is not restricted to one country. No consistent increase in risk was found in outdoor workers compared with indoor workers of similar socio-economic status.


*Br. J. Cancer* (1980) **41**, 757

### MALIGNANT MELANOMA: SOCIAL STATUS AND OUTDOOR WORK

J. A. H. LEE AND D. STRICKLAND

*From the Department of Epidemiology, School of Public Health and Community Medicine, University of Washington, Seattle, WA 98195, U.S.A.*

Received 7 November 1979 Accepted 10 January 1980

**Summary.—The incidence of, and mortality from, malignant melanoma of skin in whites are strongly influenced by socio-economic conditions. Professional and administrative workers have the highest rates of all. Clerks and salesmen have higher rates than skilled manual workers, who have higher rates than unskilled workers.**

**Women, when classified by the occupation of their husbands, show a similar relationship to social status.**

**The biases of incidence data from systems of cancer registration, and mortality data from death certificates are different, and the consistency of the data from different periods and from different populations suggests that the relationship is real.**

**The bulk of the data is from Britain, but there is sufficient from the U.S. to indicate that the effect is not restricted to one country.**

**No consistent increase in risk was found in outdoor workers compared with indoor workers of similar socio-economic status.**

THE INCIDENCE of malignant melanoma is rising rapidly (Elwood & Lee, 1975; Lee, 1976; Magnus, 1977; Teppo *et al.*, 1978; Malec & Eklund, 1978; Soodalter-Toman *et al.*, 1979) and, although prognosis is improving (End Results, 1976), this is not keeping pace, and the death rate is rising at about 3% a year (Lee *et al.*, 1979). The reason for these changes is unknown.

Malignant melanoma incidence and mortality in whites are related to latitude of residence (Lancaster, 1956; Elwood *et al.*, 1974; Magnus, 1976), duration of residence in a sunny place (Movshovitz & Modan, 1973; Anaise *et al.*, 1978) and ability to tan. The tumours select exposed anatomical sites, with particular reference to the different clothing and hair styles of men and women (Elwood & Lee, 1975; Committee, 1976). Clearly exposure to sunlight is important. However, it was shown in the data on occupational mortality of the Registrar General for England and Wales (General Register Office, 1971; Lee, 1977) that the occupational groups that suffered the highest mortality from melanomas of skin were clerical and professional workers. These typically do not conduct their business in the open air. Further data and analysis by the Registrar General extended these findings (OPCS, 1978). An increased risk of melanoma with college education and with high income was found for males in the U.S. Third National Cancer Survey, but not for females (Williams & Horm, 1977).

It is the purpose of this paper to draw together the data from a number of publications, to display in convenient form the information currently available, and to examine this in greater detail than hitherto.

### DATA AND METHODS

*Cases and deaths.*—Data on the incidence of malignant melanoma by occupation have been taken from the 1968–70 Supplement on Cancer to the Registrar General's Statistical

Review of England and Wales (OPCS, 1975). These are the latest data available. Mortality data for England and Wales were taken from the Occupational Mortality Decennial Supplements for 1951, 1961 and 1971 (GRO, 1957; 1958; 1971; OPCS, 1978). American data were derived from mortality in the State of Washington (Milham, 1976).

*Populations at risk*.—For the British mortality data, which cover periods around the decennial census, population data are taken from the appropriate census. There has been a series of methodological studies comparing the occupations recorded at the census and on the death certificate (OPCS, 1978). There are no population data for the British cancer registration data. The report comments "Because of deficiencies in statements of occupation, cancer registrations by occupation cannot be related to populations at risk" (OPCS, 1975). This is regrettable, but it should be noted that the British cancer registration system is apparently unique among the larger systems in having any usable information about this extremely potent factor in cancer aetiology. There is none, for example, in the data from the U.S. National Cancer Institute SEER programme.

*Statistical methods*.—The British mortality data have been expressed as standard mortality ratios (SMR)—*i.e.* the numbers of deaths × 100 divided by the expected numbers of deaths obtained by applying the rates for the total employed population to the population at risk of the occupation of interest. A discussion of the methodology is in the most recent report (OPCS, 1978).

The case and death data without population estimates have been analysed, as in the original reports, by proportional ratios. These compare the distribution of the total cancers for the occupation by site with the total cancers for all employed men in the British registration data (OPCS, 1975) or this distribution compared with the distribution of all causes for the employed men, in the Washington State data (Milham, 1976). While proportional ratios contain less information than mortality ratios because there was no information about populations at risk, and are susceptible to distortion by large differences in risks between occupations, they are useful if taken in conjunction with other information. They have been of historical importance: for example, in providing the first evidence for the carcinogenic activity of low levels of radiation in adults (Milham, 1976).

A likely source of perturbation of proportional cancer distributions is lung cancer, because of the very large number of cases and their variations with time (Stevens & Moolgavkar, 1979) and with occupation (OPCS, 1978). In the tables of proportional ratios, the corresponding lung-cancer ratios have been given.

*Occupational groupings*.—In Britain, occupations recorded on census forms and death certificates were coded to 223 *Occupation Units*—*e.g.* 079 butchers and meat cutters; 080 brewers, wine makers and related workers—using a standard classification of occupations (Central Statistical Office, 1968). In Washington State a modification of the standard U.S. classification was used (Milham, 1976).

Data for single occupations are important for identifying specific risks. But it has long been clear that much wider aspects of the way of life associated with groups of occupations have strong effects on the distribution by cause of death, and its timing. Since 1911 the Registrar General has grouped occupations into broad *Social Classes*—I, Professional; II, Intermediate, including many commercial occupations and teachers; III, Skilled Workers (divided for the 1970–72 data into IIIN non-manual and IIIM manual); IV, Semi-skilled and V Unskilled workers. This simple grouping has been of great use over the years, but it does group together in the same class people of very different educational attainments and economic levels.

A new socio-economic allocation of occupations developed in 1951 arranged the *Occupation Units* in a somewhat different way. They were assembled into *Socio-economic Groups* that were intended to contain people whose social, cultural and recreational standards and behaviour were similar. This system of socio-economic groups was not used for the 1959–63 mortality analysis, but was used, in a substantially modified form, for the 1970–72 data.

In 1961 a system of occupational grouping, bringing together complete industries into *Occupation Orders*, was introduced. This was also used for 1970–72. The general problem of grouping occupations is discussed in the 1971 reports (OPCS, 1978). There were not enough deaths for stable rates to be found for malignant melanoma for separate occupations, and the analyses presented will be restricted to occupations grouped into *Social Classes*, *Socio-economic Groups*, and *Occupation Orders*.

*Women and occupation.*—There are too few unmarried women for useful occupational rates for malignant melanoma to be calculated, and the personal occupations outside the home of married women are poorly reported on death certificates. But the occupations of the husbands of dead women are reported well, and have proved over the years to be a potent indicator of the general effects, apart from the specific hazards, of particular occupations (OPCS, 1978). Mortality of women has therefore been shown here by the *Social Class*, *Socio-economic Group*, or *Occupation Order* of their husbands.

### RESULTS

The Registrar General's *Socio-economic Classes* include the entire employed population in their 6 groups. Mortality rates for malignant melanoma for the three periods 1949–53, 1959–63 and 1970–72 are shown for males in Fig. 1. The data for the three periods are combined in Table I.

FIG. 1.—Standardized mortality ratios of mortality from malignant melanoma in three separate periods by the Registrar General's socio-economic classes. Males, England and Wales. (Data from GRO, 1957, 1971, OPCS, 1978).

TABLE I.—*Standardized mortality ratios (and numbers of deaths) for malignant melanoma by social class. Registrar General's Occupational Mortality Reports* 1949–72

| | Socio-economic class | Male | Female |
|---|---|---|---|
| V | Unskilled | 90 (121) | 88 (99) |
| IV | Partly skilled | 85 (217) | 82 (198) |
| IIIM | Skilled manual | 92 (485) | 103 (524) |
| IIIN | Skilled non-manual | 123 (192) | 116 (177) |
| II | Intermediate | 120 (290) | 118 (293) |
| I | Professional | 143 (80) | 140 (76) |

There is a general tendency for the rates to increase with progression in occupational status. The effect is visible between manual workers and non-manual (V, IV and IIIM compared with IIIN), and from clerical workers (IIIN) to professionals (II and I).

The relationship among women classified *by the occupation of their husbands* is

FIG. 2.—Standardized mortality ratios of mortality from malignant melanoma in three separate periods by the Registrar General's socio-economic classes. Married women by occupation of husband, England and Wales. (Data as for Fig. 1.)

Analysis of variance by social class and sex shows that sex accounts for little of the variance, and that the means for sex pooled across social class are virtually the same.

TABLE II.—*Standardizes mortality ratios (and numbers of deaths) for malignant melanoma* 1959–63 *and* 1970–72, *England and Wales by selected Occupation Orders*

| Occupation Order | | 1959–63 | 1970–72 |
|---|---|---|---|
| I | Farmers, etc.* | 90 (26) | 103 (20) |
| XV | Construction workers | 95 (19) | 67 (12) |
| VII | Engineering trades† | 87 (68) | 87 (64) |
| XX | Warehousemen, etc.§ | 85 (17) | 120 (21) |
| XXI | Clerical workers | 122 (49) | 112 (38) |
| XXII | Sales workers | 123 (58) | 127 (49) |
| XXIV | Administrators and managers | 115 (30) | 121 (39) |
| XXV | Professional and Technical‡ | 117 (49) | 142 (72) |

* Farmers, foresters, fishermen (includes workers and employers).

† Engineering and allied trades workers not elsewhere classified.

‡ Professional, technical workers, artists.

§ Warehousemen, storekeepers, packers, bottlers.

Age range for all Orders 15–64. (Data sources: GRO, 1971 and OPCS, 1978.)

TABLE III.—*Standardized mortality ratios (and numbers of deaths) for malignant melanoma in men and their wives where the men were employed in agriculture or construction. England and Wales*

| | 1949–53 | 1959–63 | 1970–72 |
|---|---|---|---|
| Agriculture etc.* | | | |
| Men | 77 (17) | 90 (26) | 103 (20) |
| Wives | 84 (16) | 115 (30) | 134 (25) |
| Construction | | | |
| Men | —† | 95 (19) | 67 (12) |
| Wives | — | 120 (24) | 107 (19) |

* For 1949–53, Socio-economic Groups 1 (farmers) and 2 (agricultural workers) were combined. For 1959–63 and 1970–72 Occupation Order I (farmers, foresters, and fishermen) was used.

The probability that the female SMR is less than or equal to the male in these 5 groups is less than 0·009 (paired *t* test).

† The socio-economic groups available for 1949–53 do not give construction workers separately. Social Class Va (building and dock labourers) had an SMR of 54 (7) for males, and 118 (13) for females. For 1959–63 and 1970–72, Occupation Order XV (construction workers) was used.

(Data sources GRO, 1957, 1971 and OPCS, 1978.)

identical to that among men classified by their own occupations (Table I; Fig. 2).

Data by occupations grouped into "orders" enable finer detail to be displayed, *e.g.* separating clerks and sales workers (Table II). Agricultural workers and construction workers do not spend their entire working lives in the open air, but it is reasonable to suppose that they spend more time in this way than do those in the engineering trades or who work in warehouses, packing plants, *etc.* These outdoor workers are at no disadvantage for melanoma mortality compared with those of similar status, and all these groups do better than the professionals.

Men in agricultural and construction jobs have lower risks of dying of malignant melanoma than their wives (Table III).

Incidence data are shown in Table IV for the same "occupation orders" as the mortality data in Table II. The gradient from manual indoor workers to the professionals is more marked, and the agricultural workers, but not the construction workers, show an increased incidence. There is a tendency for the proportional ratios for malignant melanoma to be higher in *Occupation Orders* where the ratios for lung cancer are low (Table IV). The numbers of lung-cancer cases make up so large a proportion of the total that some of the melanoma ratio changes will be simply passive. However, this cannot account for all of the variations in the melanoma ratios. Thus the engineering tradesmen, warehousemen and sales workers all have lung-cancer proportional ratios close to the mean for all employed men. Their melanoma proportional ratios are different, and have the same relationship to each other as the melanoma mortality ratios, which are not derived from proportions (Table II). There appears to be no need to postulate anything (such as social variations in tumour fatality, in care-seeking behaviour, *etc.*) other than variations in incidence to account for the social gradient of melanoma mortality.

It is important to establish that epidemiological relationships are valid in more than one population. Mortality has been tabulated for the State of Washington by age, cause, and occupation for white males from 1951 to 1971 (Milham, 1976). Analysis has again been by the construction of expected numbers of deaths on the assumption that the distribution of deaths by cause at each age

TABLE IV.—*Standardized proportional registration ratios (and numbers of cases) for malignant melanoma and lung cancer* 1966–67 *and* 1968–70 *by selected Occupation Orders, England and Wales*

| | Malignant melanoma | | Lung cancer | |
|---|---|---|---|---|
| Occupation Order* | 1966–67 | 1968–70 | 1966–67 | 1968–70 |
| Farmers etc. | 111 (22) | 124 (36) | 85 (1414) | 85 (2111) |
| Construction workers | 82 (14) | 81 (22) | 111 (1350) | 111 (2203) |
| Engineering trades | 91 (63) | 88 (90) | 105 (4304) | 106 (1159) |
| Warehousemen, etc. | 79 (13) | 93 (20) | 103 (1958) | 103 (1958) |
| Clerical workers | 103 (38) | 133 (69) | 89 (2256) | 88 (3321) |
| Sales workers | 132 (40) | 120 (54) | 103 (2151) | 99 (3139) |
| Administrators, etc. | 167 (29) | 176 (55) | 84 (959) | 85 (1812) |
| Professional and Technical | 166 (55) | 163 (89) | 72 (1125) | 73 (1959) |

* As in Table II. (Data source: OPCS, 1975.)

TABLE V.—*Proportionate mortality ratio (and numbers of deaths) for malignant melanoma and from lung cancer. White males, State of Washington,* 1951–71

| | Malignant melanoma | Lung cancer |
|---|---|---|
| White-collar non-farming | 132 (83) | 88 (1241) |
| Farmer | 84 (21) | 80 (507) |
| Farm worker | 40 (2) | 105 (126) |
| Blue-collar indoor | 115 (128) | 105 (2742) |
| Blue-collar outdoor (non-farming) | 95 (63) | 109 (1532) |
| Blue-collar unclassified | 88 (46) | 96 (1322) |

Typical outdoor blue-collar occupations, and their PMRs, are loggers (69) and parking and garage attendants (80). Typical indoor blue-collar occupations are bartenders (200) and plumbers and pipefitters (150). Blue-collar trades (unclassified) included carpenters (142) and labourers not otherwise classified (38). (Data source: Milham, 1976.)

was that of the deaths in all occupations. The proportionate mortality ratios for outdoor and indoor blue-collar occupations are similar and, as with the British data, indicate that outdoor work does not predispose to the development of malignant melanoma to any great extent (Table V). The mortality ratios for lung cancer have been included, and show little difference between the working groups of comparable socio-economic status.

### DISCUSSION

The size and consistency of the relationship of melanoma risks to some factors associated with better education, high social status, or more money, and the presence of the relationship in both employed men and among their wives when classified by husband's job, suggest that the effect is real.

It should be emphasized that the effect of high socio-economic status on the incidence of malignant melanoma is quite different from any specific carcinogenic effect of chemical agents met with in industry. Thus Hoover & Fraumeni (1975) found an excess mortality from melanoma in males and females in U.S. counties where there was a chemical industry. There have been a number of reports relating risk of melanoma to industrial agents such as PCBs (Bahn *et al.*, 1976) or some dusty occupations (Bross *et al.*, 1978).

The population data discussed here cover rather broad occupational groups. Further, because they cover all mortality and melanoma is not a common cause of death, the melanoma rates are based on quite small numbers. Hence they are not likely to demonstrate a specific industrial hazard when it applies to a small proportion of a total occupation. But the data are well adapted to displaying broad social trends.

The generality of the effect in both incidence and mortality, across a wide variety of occupations, and in both sexes, suggests that the relationship is a biological one between melanoma incidence and some feature of life associated with education or economic status. In the British occupational mortality tabulations (OPCS, 1978) there are increasing rates among the less skilled workers for cancers of the lung and stomach. Otherwise "there appears to be hardly any social class gradient for other cancers, as a group". There is thus no indication that the increased incidence and mortality from the melanomas among the white-collar and professional workers is an artefact of the tabulations.

There is currently no satisfactory explanation of this social gradient in the incidence of malignant melanoma. It has been suggested that polyunsaturated fatty acids in the diet were important, but this was not supported by further study (Goldrick *et al.*, 1976). Alcohol has also been suggested as a factor in the aetiology of malignant melanoma (Williams, 1976) but the relationship is not large or consistent (Lyon *et al.*, 1976). Outdoor work is clearly a minor factor, and it may well be that intermittent exposure—at the weekend or on a Mediterranean or tropical vacation—is important. But a test of this, or any other hypothesis, awaits the conduct of a range of clinical and epidemiological studies that are based on larger numbers and are more sophisticated in design than those currently reported (Gellin *et al.*, 1969; Klepp & Magnus, 1979).

The lack of excess incidence and mortality from malignant melanoma of skin in outdoor workers in Britain when compared with indoor workers of approximately equal social status, or with their wives, is interesting. Occupational selection may be important, or it may be that steady exposure in Britain is not an aetiological factor. Certainly the British climate is capable of shifting the distribution of lethal melanomas to the exposed ears of males and the legs of females (Lee & Yongchaiyudha, 1971) just as it does in sunnier places (Beardmore *et al.*, 1969).

One of the paradoxes of the epidemiology of malignant melanoma is that, although among white people the rates tend to increase with nearness of residence to the Equator (Elwood *et al.*, 1974) this is not so in Europe (Lee & Issenberg, 1972; Hakulinen *et al.*, 1978). Explanations have been suggested in terms of gradients of phenotype by latitude, with paler people living in the North, and in terms of social habits. It may well be that the relative prosperity of the northern countries is also an important factor. The inconstant difference between melanoma rates in urban and rural populations may have some of their explanation in these socioeconomic differences.

The rising incidence and mortality of malignant melanoma is due to large and consistent differences between birth cohorts as they move through life (Lee *et al.*, 1979). There is no explanation for this so far, but it is possible that the same factor that produces the social gradient in incidence is responsible for the rising rates in populations as a whole. The melanomas of the uveal tract do not share in this rise (Strickland & Lee, in preparation), and an account of their distribution by socioeconomic status of patients would be most interesting (OPCS, 1978).

This research was funded by Grants R805363 from the U.S. Environmental Protection Agency, and T32 EY 0717 from the National Institutes of Health.

### REFERENCES

ANAISE, D., STEINITZ, R. & BEN HUR, N. (1978) Solar radiation: A possible etiological factor in malignant melanoma in Israel. A retrospective study (1960–72). *Cancer*, **42**, 299.

BAHN, A. K., ROSENWAIKE, I., HERRMANN, N., GROVER, P., STELLMAN, J. & O'LEARY, K. (1976) Melanoma after exposure to PCBs. *N. Engl. J. Med.*, **295**, 450.

BEARDMORE, G. L., DAVID, N. C., MCLEOD, R., LITTLE, J. H., QUINN, R. L. & BURRY, A. F. (1969) Malignant melanoma in Queensland: A study of 219 deaths. *Aust. J. Dermatol.*, **10**, 158.

BROSS, I. D., VIADANA, E. & HOUTEN, L. (1978) Occupational cancer in men exposed to dust and other environmental hazards. *Arch. Environ. Health*, **33**, 300.

CENTRAL STATISTICAL OFFICE (1968) *Standard Industrial Classification*. 3rd ed., London, HMSO.

COMMITTEE ON IMPACTS OF STRATOSPHERIC CHANGE (1976) *Halocarbons; Environmental Effects of Chlorofluoromethane Release*. National Academy of Sciences, Washington. p. 101.

ELWOOD, J. M., LEE, J. A. H., WALTER, S. D., MO, T. & GREEN, A. E. S. (1974) Relationship of melanoma and other skin cancer mortality to latitude and ultraviolet radiation in the United States and Canada. *Int. J. Epidemiol.*, **3**, 325.

ELWOOD, J. M. & LEE, J. A. H. (1975) Recent data on the epidemiology of malignant melanoma. *Semin. Oncol.*, **2**, 149.

End Results Section (1976) *Cancer Patient Survival No.* 5. Washington: USDHEW. p. 223.

GELLIN, G. A., KOPF, A. W. & GARFINKLE, L. (1969) Malignant melanoma: A controlled study of possibly associated factors. *Arch. Dermatol.*, **99**, 43.

GENERAL REGISTER OFFICE (1957) *Registrar General's Decennial Supplement, England and Wales 1951 Occupational Mortality Part II*, Vol **2**. London: HMSO.

GENERAL REGISTER OFFICE (1958) *Registrar General's Decennial Supplement, England and Wales 1951 Occupational Mortality Part II*, Vol. **1**. London: HMSO.

GENERAL REGISTER OFFICE (1971) *Registrar General's Decennial Supplement, England and Wales 1961 Occupational Mortality*. London: HMSO.

GOLDRICK, R. B., GOODWIN, R. M., NESTEL, P. J., DAVIS, N. C., POYSER, A. & QUINLIVAN, N. L. (1976) Do polyunsaturated fats predispose to malignant melanoma? *Med. J. Aust.*, **i**, 987.

HAKULINEN, T., TEPPO, L. & SAXEN, E. (1978) Cancer of the eye: A review of trends and differentials. *Wld. Hlth. Stat. Reps.*, **31**, 143.

HOOVER, R. & FRAUMENI, J. F. (1975) Cancer mortality in U.S. counties with chemical industries. *Environ. Res.*, **9**, 196.

KLEPP, O. & MAGNUS, K. (1979) Some environmental and bodily characteristics of melanoma patients: A case-control study. *Int. J. Cancer*, **23**, 482.

LANCASTER, H. O. (1956) Some geographical aspects of the mortality from melanoma in Europeans. *Med. J. Aust.*, **1**, 1082.

LEE, J. A. H. (1976) The cuirent rapid increase in incidence and mortality from malignant melanoma in developed societies. In *Pigment Cell, Vol.* 2, Ed. Riley. Basel: Karger. p. 414.

LEE, J. A. H. (1977) Current evidence about the causes of malignant melanoma in progress. In *Clinical Cancer*, Ed. Ariel. New York: Grune & Stratton. p. 151.

LEE, J. A. H. & ISSENBERG, H. J. (1972) A comparison between England and Wales and Sweden in the incidence and mortality of malignant skin tumours. *Br. J. Cancer*, **26**, 59.

LEE, J. A. H., PETERSEN, G. R., STEVENS, R. G. & VESANEN, K. (1979) The influence of age, year of birth, and date on mortality from malignant melanoma in the populations of England and Wales, Canada and the white population of the United States. *Am. J. Epidemiol.*, **110**, 734.

LEE, J. A. H. & YONGCHAIYUDHA, S. (1971) Incidence and mortality from malignant melanoma by anatomical site. *J. Natl Cancer Inst.*, **47**, 253.

LYON, J. L., GARDNER, J. W. & KLAUBER, M. R. (1976) Alcohol and cancer. *Lancet*, **1**, 1243.

MAGNUS, K. (1977) Incidence of malignant melanoma of the skin in the five Nordic countries: Significance of solar radiation. *Int. J. Cancer*, **20**, 477.

MAGNUS, K. (1976) Epidemiology of malignant melanoma of the skin in Norway with special reference to the effect of solar radiation. In *Biological Characterisation of Human Tumours*. Eds. Davis & Maltoni. Amsterdam: Excerpta Medica. p. 249.

MALEC, E. & EKLUND, G. (1978) The changing incidence of malignant melanoma of the skin in Sweden, 1959–68. *Scand. J. Plast. Reconstr. Surg.*, **12**, 19.

MILHAM, S. (1976) *Occupational mortality in Washington State*, Washington: USDHEW.

MOVSHOVITZ, M. & MODAN, B. (1973) Role of sun exposure in the etiology of malignant melanoma: Epidemiological inference. *J. Natl Cancer Inst.*, **51**, 777.

OFFICE OF POPULATION CENSUSES AND SURVEYS (1975) *The Registrar General's Statistical Review of England and Wales for the three years 1698–70, Supplement on Cancer*. London: HMSO.

OFFICE OF POPULATION CENSUSES AND SURVEYS (1978) *Occupational Mortality, The Registrar General's Decennial Supplement for England and Wales, 1970–72.* Series DS, no. 1, London: HMSO.

REGISTRAR GENERAL (1975) *Statistical Review of England and Wales for the three years 1968–70, Supplement on Cancer*. London: HMSO. p. 217.

SOODALTER-TOMAN, D. L., WEST, D. W. & DERRICK, L. R. (1980) Epidemiology of malignant melanoma in Utah 1966–76.

STEVENS, R. G. & MOOLGAVKAR, S. H. (1979) Estimation of relative risk from vital data: Smoking and cancers of the lung and bladder. *J. Natl Cancer Inst.*, **631**, 1351.

TEPPO, L., PAKKANEN, M. & HAKULINEN, T. (1978) Sunlight as a risk factor of malignant melanoma of the skin. *Cancer*, **41**, 2018.

WILLIAMS, R. R. (1976) Breast and thyroid cancer and malignant melanoma promoted by alcohol-induced pituitary secretion of prolactin T.S.H. and M.S.H. *Lancet*, i, 1243.

WILLIAMS, R. R. & HORM, J. W. (1977) Association of cancer sites with tobacco and alcohol consumption and socioeconomic status of patients. Interview study from the Third National Cancer Survey. *J. Natl Cancer Inst.*, **58**, 525.